# Identifying Drug-Induced Liver Injury Associated With Inflammation-Drug and Drug-Drug Interactions in Pharmacologic Treatments for COVID-19 by Bioinformatics and System Biology Analyses: The Role of Pregnane X Receptor

**DOI:** 10.3389/fphar.2022.804189

**Published:** 2022-08-01

**Authors:** Jingjing Huang, Zhaokang Zhang, Chenxia Hao, Yuzhen Qiu, Ruoming Tan, Jialin Liu, Xiaoli Wang, Wanhua Yang, Hongping Qu

**Affiliations:** ^1^ Department of Pharmacy, Ruijin Hospital, Shanghai Jiaotong University School of Medicine, Shanghai, China; ^2^ Department of Pharmacy, Shanghai Children’s Medical Center, Shanghai Jiaotong University School of Medicine, Shanghai, China; ^3^ Department of Critical Care, Ruijin Hospital, Shanghai Jiaotong University School of Medicine, Shanghai, China

**Keywords:** drug induced liver injury, COVID-19, SARS-CoV-2, inflammation-drug interactions, drug-drug interactions, PXR

## Abstract

Of the patients infected with coronavirus disease 2019 (COVID-19), approximately 14–53% developed liver injury resulting in poor outcomes. Drug-induced liver injury (DILI) is the primary cause of liver injury in COVID-19 patients. In this study, we elucidated liver injury mechanism induced by drugs of pharmacologic treatments against SARS-CoV-2 (DPTS) using bioinformatics and systems biology. Totally, 1209 genes directly related to 216 DPTS (DPTSGs) were genes encoding pharmacokinetics and therapeutic targets of DPTS and enriched in the pathways related to drug metabolism of CYP450s, pregnane X receptor (PXR), and COVID-19 adverse outcome. A network, constructed by 110 candidate targets which were the shared part of DPTSGs and 445 DILI targets, identified 49 key targets and four Molecular Complex Detection clusters. Enrichment results revealed that the 4 clusters were related to inflammatory responses, CYP450s regulated by PXR, NRF2-regualted oxidative stress, and HLA-related adaptive immunity respectively. In *cluster 1*, IL6, IL1B, TNF, and CCL2 of the top ten key targets were enriched in COVID-19 adverse outcomes pathway, indicating the exacerbation of COVID-19 inflammation on DILI. PXR-CYP3A4 expression of *cluster 2* caused DILI through inflammation-drug interaction and drug-drug interactions among pharmaco-immunomodulatory agents, including tocilizumab, glucocorticoids (dexamethasone, methylprednisolone, and hydrocortisone), and ritonavir. NRF2 of *cluster 3* and HLA targets of *cluster four* promoted DILI, being related to ritonavir/glucocorticoids and clavulanate/vancomycin. This study showed the pivotal role of PXR associated with inflammation-drug and drug-drug interactions on DILI and highlighted the cautious clinical decision-making for pharmacotherapy to avoid DILI in the treatment of COVID-19 patients.

## Introduction

Coronavirus disease 2019 (COVID-19) caused by severe acute respiratory syndrome coronavirus 2 (SARS-CoV-2) is continuing to spread globally. This disease resulted in thousands of hospitalizations and deaths (Galloway et al., 2021; Hu et al., 2021). As per studies, 14–53% of COVID-19 patients developed different degrees of liver injuries resulting in poor outcomes, especially critically ill patients with the cytokine release syndrome (CRS) (Jothimani et al., 2020; Abou-Arab et al., 2021). It has been reported that drug-induced liver injury (DILI) is the primary cause of liver injury in COVID-19 patients, besides SARS-CoV-2 and the preexisting primary liver diseases (Papadopoulos et al., 2020). DILI is one of the most hazardous adverse drug reactions and results in liver injury, acute liver failure, liver transplantation, or even death (Reuben et al., 2010). Furthermore, COVID-19 is a systemic disease that can present many complications, including liver injury as result of CRS in severe COVID-19 infection. This liver disorder would increase the susceptibility to DILI in the pharmacotherapy of COVID-19 patients (Sodeifian et al., 2021).

In addition, drugs of pharmacologic treatments against SARS-CoV-2 (DPTS) are repurposed and off-label used in combination due to the lack of standard guideline for COVID-19 management (Ortiz et al., 2021). Pharmacotherapy against SARS-CoV-2 not only inhibits virus replication, but also mediates immune-induced inflammation in COVID-19 patients suffering this specific disease state (both virus infection and CRS) (Yongzhi, 2021). DPTS mainly include antiviral drugs and pharmaco-immunomodulatory agents, e.g., lopinavir/ritonavir and tocilizumab (Rizk et al., 2020; Singh et al., 2020). Furthermore, additional drugs to treat other complications such as thrombosis or bacterial co-infection are also administrated to COVID-19 patients hospitalized or admitted to intensive care unit (ICU) (Cattaneo et al., 2020; Magro, 2020; Manohar et al., 2020; Ali and Spinler, 2021). Polypharmacy contributes to the increased risk of DILI due to drug-drug interactions (DDIs). It has been reported that DDIs modified drug hepatic safety in the administration of co-medications (Suzuki et al., 2015). In a large-scale study of COVID-19 hospitalized patients, co-medications were found to be associated with a six-fold increased risk of DILI (Iloanusi et al., 2021). DILI is triggered by hepatic metabolism (pharmacokinetics, PK) of drugs: phase I metabolism of lipophilic drugs, via drug metabolizing enzymes (DMEs) - CYP450s in the liver, generates reactive oxidative metabolites. The covalent binding of metabolites leads to an immune response (metabolic idiosyncratic DILI) or ROS release with glutathione (GSH) depletion (oxidative stress) in mitochondria and endoplasmic reticulum, directly causing hepatic necrosis or apoptosis (intrinsic DILI) (European Association for the Study of the Liver, 2019).

Accumulating evidences (Awortwe and Cascorbi, 2020; Ferron et al., 2020; Hodge et al., 2020) have showed that PK DDIs, primarily related to the presence of CYP3A4, might contribute to DILI in COVID-19 patients. Because most of the repurposed drugs against COVID-19, such as lopinavir/ritonavir, are the substrates of CYP3A4 (Sanders et al., 2020). CYP3A4, involved in the metabolism of over 50% of clinically used drugs, is highly sensitive to the pregnane X receptor (PXR)’s upstream modulation. CYP450s such as 3A, as well as drug transporters of UDP-Glucuronosyltransferases (UGTs) and P-glycoprotein (P-gp), are transcriptionally regulated by the nuclear receptor of PXR that is encoded by the nuclear receptor subfamily 1, group I, member 2 (NR1I2) gene (Cheng et al., 2011). PXR-regulated drug metabolism (DM), mainly associated with CYP3A4, was involved in the DILI in the pharmacologic treatments against SARS-CoV-2 (El-Ghiaty et al., 2020). During COVID-19 infection, PXR inhibition by the overwhelming released cytokines [Interleukin (IL)-6, Tumour Necrosis Factor (TNF)-ɑ, and IL-1β] via NF-κB caused the impaired DM and inflammation-drug interactions, accompanying with the increased DILI risk (Lv and Huang, 2020). PXR interacting with NF-κB provides a potential molecular mechanism that links CRS of COVID-19 and hepatic metabolism of DPTS. On the other hand, PXR activation mediated by interactions between pharmaco-immunomodulatory agent of tocilizumab and ritonavir can cause the metabolic DILI due to the increased production of CYP3A4-induced reactive metabolites of ritonavir (Shehu et al., 2019; Muhović et al., 2020). Ritonavir, commonly used with lopinavir against SARS-CoV-2, is a potent regulator of PXR and CYP3A4 (Cao et al., 2020). However, ritonavir comes with a black box warning from the United States Food and Drug Administration due to an increased probability of fatal DDIs. A clinical study of 417 hospitalized COVID-19 patients showed that the use of lopinavir/ritonavir could significantly lead to a higher risk of liver injury (OR 5.03, 95% CI 1.78–14.23; *p* < 0.01) (Cai et al., 2020). Moreover, certain drugs including lopinavir/ritonavir and tocilizumab were responsible for DILI in COVID-19 patients (Boeckmans et al., 2020; Olry et al., 2020; Zhang et al., 2020). Currently, liver injury induced by DPTS, related with the different PK behaviors of DPTS from those provided by drug labels and the possible adverse drug combinations, are getting concern in the management of COVID-19 (Ali, 2020; Ali and Hossain, 2020; Ortiz et al., 2021; Zhang et al., 2022). However, the exact mechanism underlying DILI in COVID-19 patients were not yet fully understood and no systematic analysis exists. Here we hypothesized that PXR regulation, mediated by inflammation-drug interactions and DDIs during CRS of COVID-19, was associated with DILI.

**FIGURE 1:**
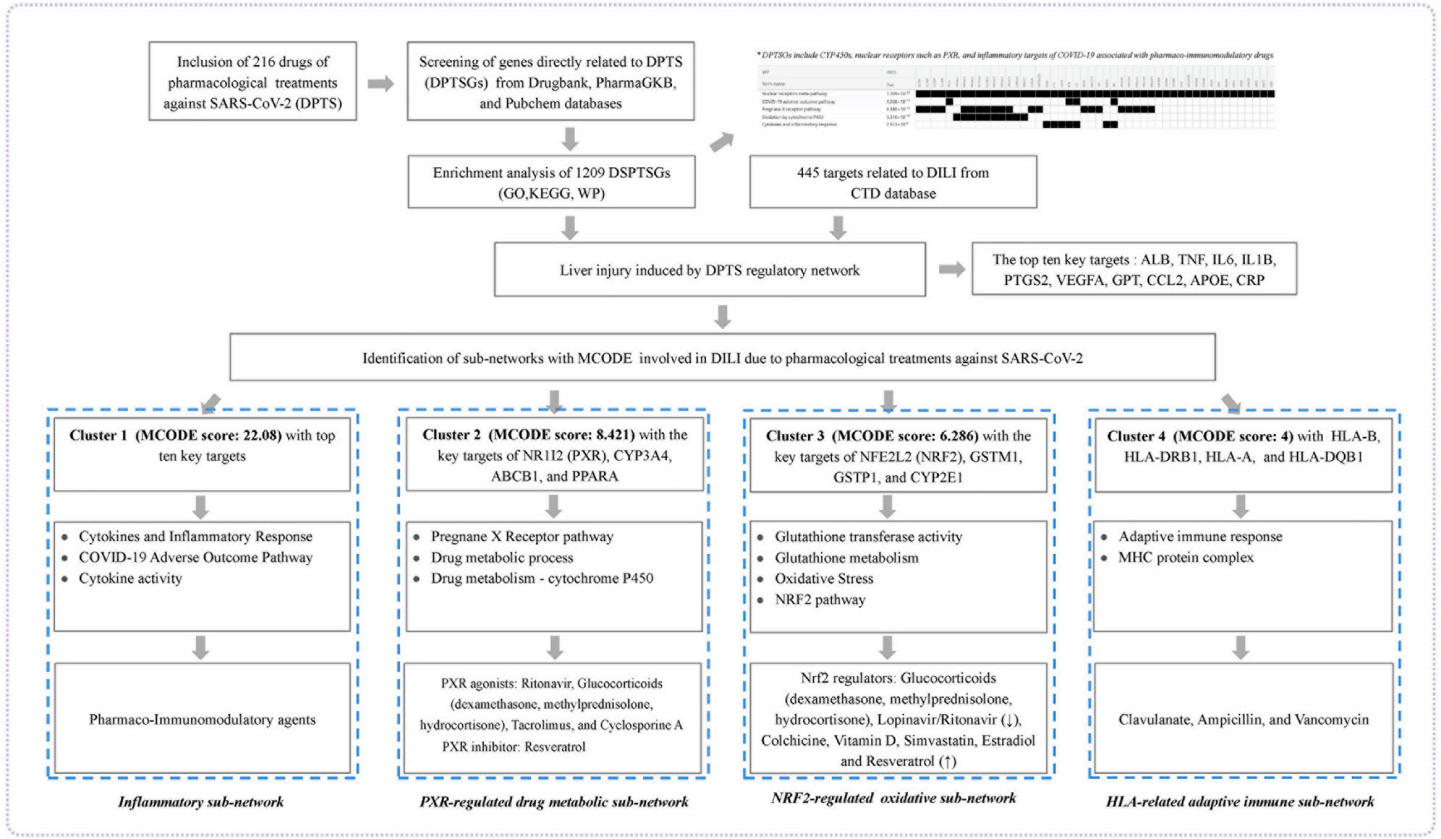
Workflow of the present study. The figure indicated the mechanism underlying liver injury induced by DPTS using bioinformatics and system biology analyses.

In this present study, we used publicly available data to comprehensively screen DPTS and genes encoding their therapeutic and PK targets (DMEs and drug transporters) named as genes that were directly related to DPTS (DPTSGs). DPTSGs were analyzed by functional enrichment and matched with DILI targets to identify candidate targets of DPTS-induced liver injury to establish regulatory network. Further MOCDE clusters analysis of candidate targets was conducted to elucidate the molecular mechanism underlying the DILI in COVID-19 patients (Figure 1).

## Methods

### Included DPTS associated with the treatment of COVID-19 patients without the primary diseases

The off-label drugs against SARS-CoV-2 were extracted from the list of COVID-19 agent section on the University of Liverpool COVID-19 drug interaction website (www.covid19-druginteractions.org) (University of Liverpool, 2022), the guidelines, random clinical trials, and literature before 31 December 2021. The searched keywords were “COVID-19”, “SARS-CoV-2”, and “2019 novel coronavirus” in English. In these data, the following studies were excluded: 1) studies that investigated non-pharmacological interventions, including cell therapies, vaccines, and convalescent plasma; 2) studies that investigated traditional Chinese medicine due to the heterogeneous nature of its drugs used and the uncertainty of active ingredients; 3) studies that included patients who were concomitant with the primary diseases. Off-label drugs obtained from the systematic searches which were conducted independently by all authors using the keywords “drug”,” therapeutic”, “treatment”, “therapy”, and “guidelines” were DPTS.

### Screening of DPTSGs and Targets of DILI

DPTSGs were retrieved from the “Targets, Enzymes, Carriers, Transporters” sections in the Drugbank database (https://go.drugbank.com/) (Wishart et al., 2018), “Variant Annotations” section in the PharmaGKB database (https://www.pharmgkb.org/) (Whirl-Carrillo et al., 2021), and “Biomolecular Interactions and Pathways” section in the Pubchem database (https://pubchem.ncbi.nlm.nih.gov/) (Kim et al., 2021). The DILI targets were obtained from the Comparative Toxicogenomics Database (http://ctdbase.org/) (Davis et al., 2021).

### Gene Ontology (GO), Kyoto Encyclopedia of Genes and Genomes (KEGG), and Wikipathway (WP) Enrichment Analysis of DPTSGs

The DPTSGs were functionally enriched in Gene Ontology (GO) [Biological Process (BP), Molecular Function (MF), and Cellular Component (CC)], Kyoto Encyclopedia of Genes and Genomes (KEGG), and Wikipathway (WP) enrichment pathways using go: profiler (https://biit.cs.ut.ee/gprofiler/gost) (Raudvere et al., 2019) to analyze the biological processes related to the therapeutic and the PK targets of DPTS (Kelder et al., 2012). The significantly enriched pathways and GO terms (Benjamini adjusted *p*-value <0.05, number of enrichment genes ≥2) were identified. The enrichments were visualized using a dot plot using the ggplot2 package in R software.

### Construction and MCODE Sub-networks Analysis of Regulatory Network of DPTS-Induced Liver Injury, Enrichment Analysis of Sub-networks Targets

The candidate targets underlying DPTS-induced liver injury were obtained by matching DPTSGs and DILI targets. The protein-protein interaction (PPI) network of candidate targets was constructed. The PPI data with medium confidence interaction score >0.4 were downloaded from the STRING database (https://cn.string-db.org/) (Szklarczyk et al., 2021), and the PPI network was visualized in Cytoscape software (https://cytoscape.org/, version 3.7.2) (Shannon et al., 2003). The nodes of this network represented proteins, and the edges represented the interactions between the two proteins. The average degree of the nodes was calculated using the Network Analyzer plugin. The targets having degrees higher than the average value were defined as the key targets. Furthermore, to identify the core local sub-networks (*clusters*) with the high clustering scores, we conducted Molecular Complex Detection (MCODE) cluster analysis of candidate targets using the Cytoscape plugin MCODE clustering algorithm (node score cut-off value = 0.2, degree cut-off value = 2, K-core = 2, MAX depth value = 100) (Zhang et al., 2019; Ahmed, 2020). The higher the clustering scores were, the more important the MOCDE sub-networks were in the regulatory network of DPTS-induced liver injury. The targets of MOCDE sub-networks were further analyzed by GO, KEGG, and WP pathways to understand the biological processes and significant pathways involved in DPTS-induced liver injury.

### Identification of Drugs Related to the DILI Targets of MOCDE Sub-networks and Its Significant Pathways

Drugs that relate to the key targets and significant pathways in each MOCDE *clusters* have a higher risk of DILI. Moreover, drugs with log *p* ≥ 3 and a daily dose ≥100 mg were associated with a significantly increased risk of DILI (*p* < 0.05) (McEuen et al., 2017). Information about the administration route and doses of the potential drugs were collected. The lipophilicity parameters (measured by log value of octanol-water partition coefficient, Log *p* values) were extracted from the “Chemical and Physical Properties” section in the Pubchem database (https://pubchem.ncbi.nlm.nih.gov/) (Kim et al., 2021). The metabolizing organ of the drugs, likelihood score of DILI, and DILI phenotypes were respectively obtained from the Drugbank database (https://go.drugbank.com/) (Wishart et al., 2018) and LiverTox: Clinical and Research Information on Drug-Induced Liver Injury [Internet] (https://www.ncbi.nlm.nih.gov/books/NBK548710/) (Hoofnagle et al., 2013), respectively.

## Results

### DPTS Drugs

**FIGURE 2:**
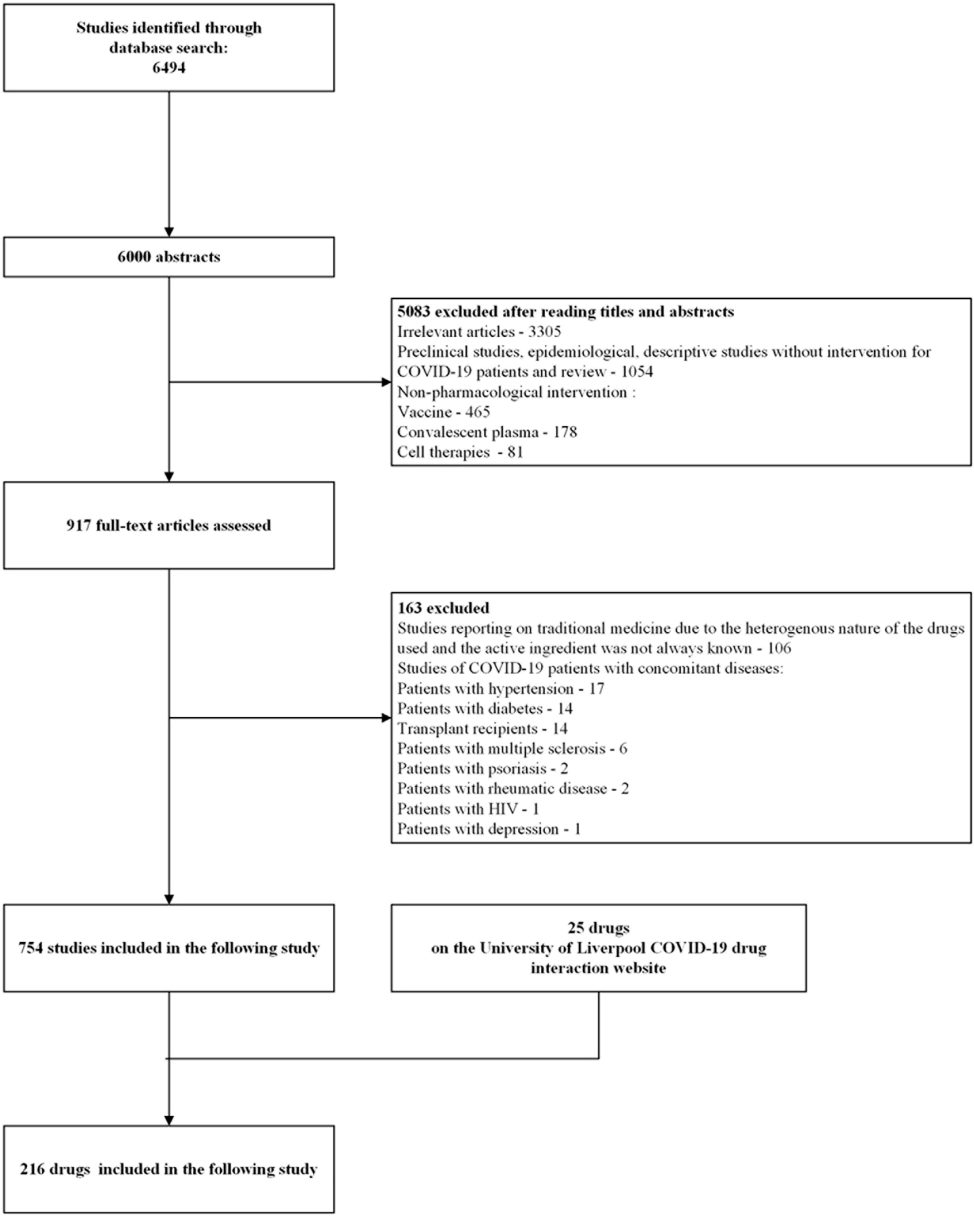
Flowchart of drugs of pharmacologic treatment against SARS-CoV-2 inclusion.

A total of 216 drugs, including antiviral drugs for SARS-CoV-2, pharmaco-immunomodulatory drugs, anticoagulants, anti-platelet drugs, and antibiotics against bacterial co-infections with SARS-CoV-2 in community and ward, were DPTS and listed in Supplementary Material. DPTS were extracted from 754 studies and the University of Liverpool COVID-19 drug interaction website (25 drugs), as shown in Figure 2.

### DPTSGs associated with biological processes of “response to chemical” and pathways of “Drug metabolism - cytochrome P450”, “nuclear receptors meta-pathway”, “pregnane X receptor pathway”, and “COVID-19 adverse outcome pathway”

**FIGURE 3:**
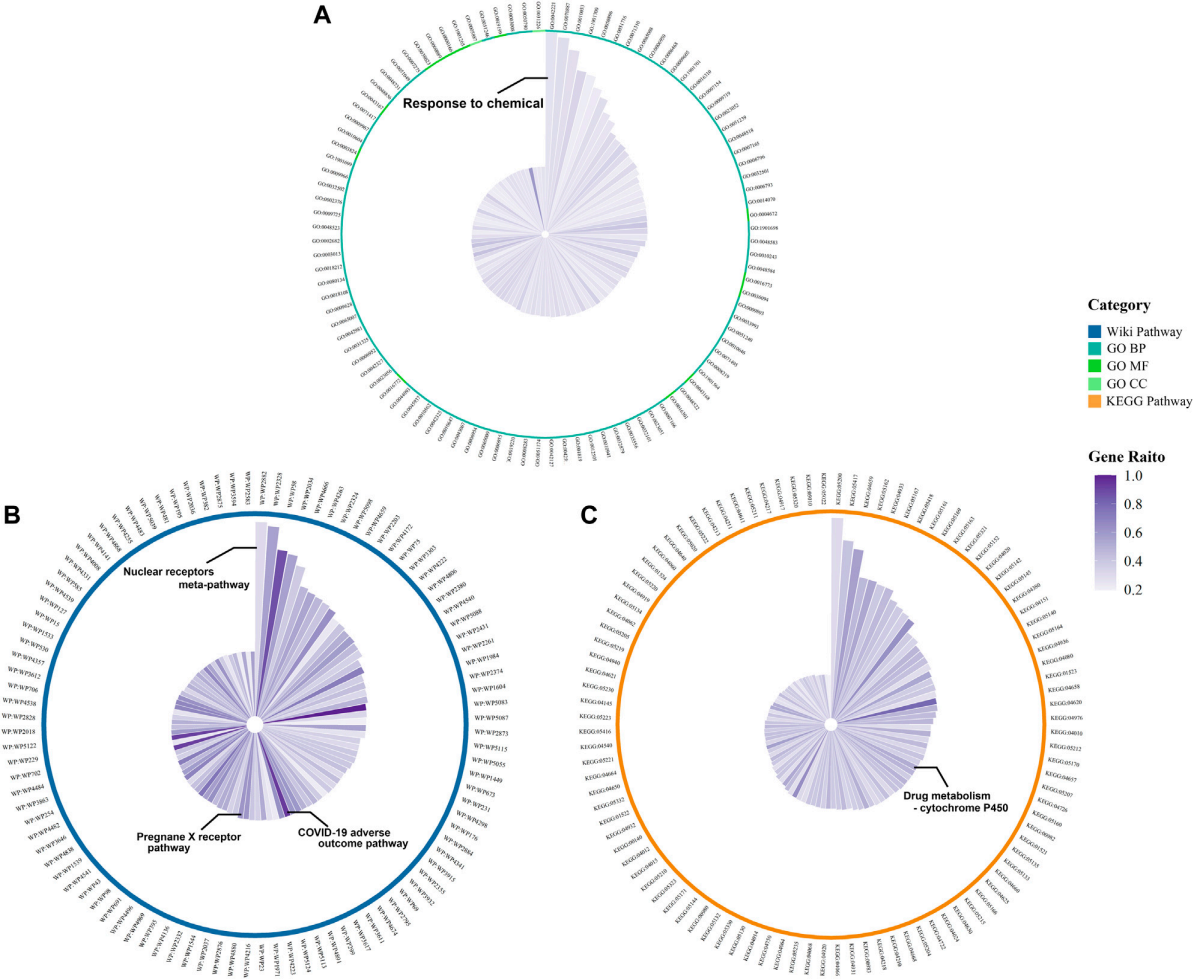
Functional enrichment results of DPTSGs: Top 100 GO terms **(A)**, KEGG pathways **(B)**, and WP pathways **(C)** (The radius of the circle is the -log_10_ *p* value of the term).

We obtained 1209 DPTSGs after deleting the duplicate genes (Supplementary Material). GO, KEGG, and WP analysis results of DPTSGs showed that DPTSGs were involved in 5935 GO terms, 204 KEGG pathways, and 406 WP pathways. The top 100 GO terms, KEGG pathways, and WP pathways with the -log_10_ *p* values were shown in Figure 3. The important biological processes of “response to chemical” (*p* = 2.16 × 10^–218^) confirmed that DPTSGs mainly included the PK targets of DPTS (I, Ⅱ phase DMEs, and Ⅲ phase drug transporters). As per the main KEGG pathway - “Drug metabolism - cytochrome P450” (*p* = 1.60 × 10^–14^), CYP450s were the main DMEs of DPTS. Furthermore, the primary WP pathway of “Nuclear Receptors Meta-Pathway” (*p* = 1.21 × 10^–23^) suggested that PK targets of DPTS were regulated by nuclear receptors, including PXR/NR1I2 (WP2876: Pregnane X Receptor pathway with *p* = 6.38 × 10^–11^). Above enrichment analysis results of DPTSGs indicated the DILI of DPTS associated with PXR-mediated CYP450s DM and the necessity of further analysis for DPTS-induced liver injury. ACE2, AGT, CCL2, CCL3, CSF3, CXCL10, CXCL8, IL10, IL1B, IL2, IL2RA, IL6, TMPRSS2, and TNF were the genes related to the “COVID-19 adverse outcome pathway” [WP4891 with *p* = 3.27 × 10^–11^ and genes ratio of 93.3% (14/15)]. The genes, including IL6, IL1B, and TNF, encode inflammatory cytokines of CRS in COVID-19. They were also the therapeutic targets of pharmaco-immunomodulatory drugs.

### Regulatory Network and Four Local Sub-networks (*Clusters* 1, 2, 3, and 4) of DPTS-Induced Liver Injury

**FIGURE 4:**
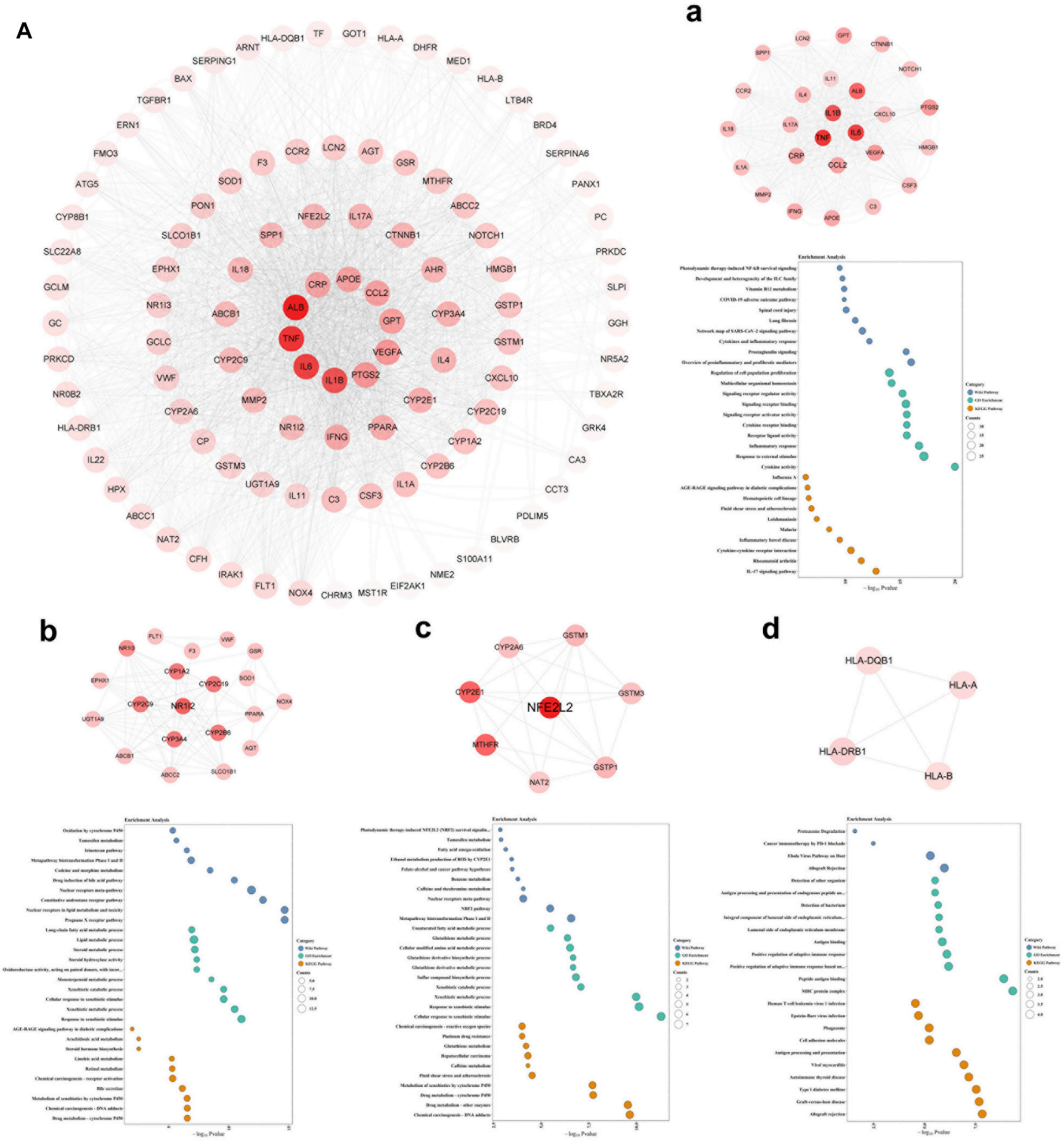
DPTS-induced liver injury regulatory network **(A)** and MCODE clusters with their top 10 GO, KEGG, and WP pathways functional enrichment results. *Cluster 1* **(a)** *, cluster 2* **(b)** *, cluster 3* **(c)** *, and cluster 4* **(d)**. GO, gene ontology; BP, biological processes; MF, molecular function; CC, cell component; KEGG, kyoto encyclopedia of genes and genomes; WP, wikipathway.

A total of 110 candidate targets from 1209 DPTSGs and 445 DILI targets were used to construct the PPI network (DPTS-induced liver injury regulatory network). The PPI network had 104 nodes (after removing six isolated candidate targets) and 1914 edges, with an average degree of 18.4 (Figure 4A). A total of 49 key targets were identified, including the top ten targets - ALB, TNF, IL6, IL1B, PTGS2, VEGFA, GPT, CCL2, APOE, and CRP. Four different local sub-networks were obtained from this network using the Cytoscape plug-in MCODE (Table 1). Among the sub-networks, *cluster 1* with the highest MCODE score of 22.08 contained the top ten key targets with high connectivity in the PPI network (Figure 4A). In the *cluster 2* sub-network (MCODE score = 8.421)*,* nuclear receptors of NR1I2 (PXR) and PPARA and their downstream CYP3A4 and ABCB1 were included (Figure 4B). The essential xenobiotic sensor PXR regulated CYP3A and P-gp (ABCB1), and PPARA had a positive influence on CYP3A expression (Klein et al., 2012). The transcription factor NFE2L2 (NRF2) with CYP2E1 manipulated the target genes of GSTM1 and GSTP1 in the *cluster 3* sub-network (Figure 4C). Identified as the HLA-sub-network, *cluster four* was involved in DILI associated with immunological reactions (Figure 4D).

**TABLE 1:** MCODE clusters and their associated genes from DPTS-induced liver injury regulatory network.

Clusters	Scores	Nodes	Edges	Node IDs
1	22.08	26	522	IFNG, CCL2, CCR2, MMP2, VEGFA, IL17A, GPT, LCN2, TNF, SPP1, ALB, IL1A, IL6, CXCL10, HMGB1, IL1B, APOE, CRP, PTGS2, IL18, CSF3, IL4, C3, CTNNB1, IL11, NOTCH1
2	8.421	20	80	CYP3A4, SOD1, PPARA, AGT, FLT1, NR1I2, F3, CYP2B6, EPHX1, CYP2C9, VWF, SLCO1B1, CYP2C19, ABCB1, NOX4, ABCC2, UGT1A9, CYP1A2, GSR, NR1I3
3	6.286	8	22	GSTM1, GSTP1, CYP2A6, NFE2L2, CYP2E1, GSTM3, MTHFR, NAT2
4	4	4	6	HLA-B, HLA-DRB1, HLA-A, HLA-DQB1

### Inflammatory Sub-network (*Cluster 1*), PXR-Regulated Drug Metabolic Sub-network (*Cluster 2*), NRF2-Regulated Oxidative Sub-network (*Cluster 3*), and HLA-Related Adaptive Immune Sub-network (*Cluster 4*)

Enrichment results of *cluster 1* revealed that this primary sub-network was associated with “cytokine activity” (*p* = 9.12 × 10^–21^), “Cytokines and Inflammatory Response” (*p* = 5.78 × 10^–13^), and “COVID-19 adverse outcome pathway” (*p* = 1.16 × 10^–10^, Table 2). Noteworthily, IL6, IL1B, TNF, and CCL2 in the COVID-19 adverse outcome pathway were found to be the DILI targets, indicating exacerbation of these inflammatory factors of CRS on DILI. The DILI targets in *cluster 2* were significantly related to drug metabolism- CYP450s regulated by PXR**: “**drug metabolic process” (*p* = 2.71 × 10^–7^), “Pregnane X Receptor pathway” (*p* = 2.10 × 10^–15^), “Nuclear Receptors in Lipid Metabolism and Toxicity” (*p* = 2.10 × 10^–15^), and “Drug metabolism - cytochrome P450” (*p* = 2.71 × 10^–7^; Table 3). In addition, *cluster 3* was a NRF2-regulated oxidative sub-network, involved in oxidative stress and GSH-glutathione transferase (GST) metabolism. HLA-B, HLA-DRB1, HLA-A, and HLA-DQB1 of *cluster four* were enriched in the MHC protein complex and involved in the regulation of adaptive immune response.

**TABLE 2:** Enrichment analysis results of *cluster 1* genes (Top 10 biological functions, WP pathways, and KEGG pathways).

ID	GO term/Pathway Description	Source	*p* Value	Associated Genes (%)	Number of Genes
GO:0005125	cytokine activity	GO:MF	9.12 × 10^–21^	6.38	15
GO:0009605	response to external stimulus	GO:MF	6.10 × 10^–18^	0.91	25
GO:0006954	inflammatory response	GO:MF	1.74 × 10^–17^	2.38	18
GO:0048018	receptor ligand activity	GO: BP	2.23 × 10^–16^	3.03	15
GO:0005126	cytokine receptor binding	GO:MF	2.23 × 10^–16^	4.71	13
GO:0030546	signaling receptor activator activity	GO: BP	2.23 × 10^–16^	2.99	15
GO:0005102	signaling receptor binding	GO:MF	2.70 × 10^–16^	1.29	20
GO:0030545	signaling receptor regulator activity	GO: BP	5.50 × 10^–16^	2.74	15
GO:0048871	multicellular organismal homeostasis	GO: BP	5.46 × 10^–15^	2.85	15
GO:0042127	regulation of cell population proliferation	GO: BP	8.83 × 10^–15^	1.21	20
WP: WP5095	Overview of proinflammatory and profibrotic mediators	WP	9.02 × 10^–17^	11.02	14
WP: WP5088	Prostaglandin signaling	WP	2.57 × 10^–16^	30.30	10
WP: WP530	Cytokines and inflammatory response	WP	5.78 × 10^–13^	30.77	8
WP: WP5115	Network map of SARS-CoV-2 signaling pathway	WP	2.49 × 10^–12^	5.88	13
WP: WP3624	Lung fibrosis	WP	1.13 × 10^–11^	14.29	9
WP: WP2431	Spinal cord injury	WP	7.68 × 10^–11^	8.47	10
WP: WP4891	COVID-19 adverse outcome pathway	WP	1.16 × 10^–10^	40.00	6
WP: WP1533	Vitamin B12 metabolism	WP	1.16 × 10^–10^	15.09	8
WP: WP3893	Development and heterogeneity of the ILC family	WP	1.68 × 10^–10^	21.88	7
WP: WP3617	Photodynamic therapy-induced NF-kB survival signaling	WP	2.99 × 10^–10^	20.00	7
KEGG:04657	IL-17 signaling pathway	KEGG	1.44 × 10^–13^	11.96	11
KEGG:05323	Rheumatoid arthritis	KEGG	3.21 × 10^–12^	11.36	10
KEGG:04060	Cytokine-cytokine receptor interaction	KEGG	2.79 × 10^–11^	4.44	13
KEGG:05321	Inflammatory bowel disease	KEGG	2.92 × 10^–10^	12.90	8
KEGG:05144	Malaria	KEGG	2.70 × 10^–9^	14.29	7
KEGG:05140	Leishmaniasis	KEGG	3.68 × 10^–8^	9.72	7
KEGG:05418	Fluid shear stress and atherosclerosis	KEGG	1.13 × 10^–7^	5.80	8
KEGG:04640	Hematopoietic cell lineage	KEGG	1.98 × 10^–7^	7.37	7
KEGG:04933	AGE-RAGE signaling pathway in diabetic complications	KEGG	2.52 × 10^–7^	7.00	7
KEGG:05164	Influenza A	KEGG	3.76 × 10^–7^	4.76	8

**TABLE 3:** Enrichment analysis results of *cluster 2* genes (Top 10 biological functions, WP pathways, and KEGG pathways).

Id	GO term/Pathway Description	Source	*p* Value	Associated Genes, %	Number of Genes
GO:0009410	response to xenobiotic stimulus	GO: BP	8.36 × 10^–12^	4.13%	10
GO:0006805	xenobiotic metabolic process	GO: BP	3.05 × 10^–11^	7.48%	8
GO:0071466	cellular response to xenobiotic stimulus	GO: BP	2.54 × 10^–10^	5.30%	8
GO:0042178	xenobiotic catabolic process	GO: BP	2.54 × 10^–10^	17.14%	6
GO:0016098	monoterpenoid metabolic process	GO: BP	2.64 × 10^–9^	66.67%	4
GO:0016712	oxidoreductase activity, acting on paired donors, with incorporation or reduction of molecular oxygen, reduced flavin or flavoprotein as one donor, and incorporation of one atom of oxygen	GO:MF	4.40 × 10^–8^	12.50%	5
GO:0008395	steroid hydroxylase activity	GO:MF	4.40 × 10^–8^	13.51%	5
GO:0008202	steroid metabolic process	GO: BP	6.49 × 10^–8^	2.52%	8
GO:0006629	lipid metabolic process	GO: BP	7.37 × 10^–8^	0.89%	12
GO:0001676	long-chain fatty acid metabolic process	GO: BP	1.14 × 10^–7^	5.50%	6
WP: WP2876	Pregnane X receptor pathway	WP	2.10 × 10^–15^	27.27	9
WP: WP299	Nuclear receptors in lipid metabolism and toxicity	WP	2.10 × 10^–15^	25.71	9
WP: WP2875	Constitutive androstane receptor pathway	WP	1.35 × 10^–13^	25.00	8
WP: WP2882	Nuclear receptors meta-pathway	WP	1.22 × 10^–12^	4.05	13
WP: WP2289	Drug induction of bile acid pathway	WP	3.25 × 10^–11^	35.29	6
WP: WP1604	Codeine and morphine metabolism	WP	3.42 × 10^–9^	33.33	5
WP: WP702	Metapathway biotransformation Phase I and II	WP	1.30 × 10^–7^	4.32	8
WP: WP229	Irinotecan pathway	WP	2.98 × 10^–7^	30.77	4
WP: WP691	Tamoxifen metabolism	WP	2.19 × 10^–6^	19.05	4
WP: WP43	Oxidation by cytochrome P450	WP	4.45 × 10^–6^	7.94	5
KEGG:00982	Drug metabolism - cytochrome P450	KEGG	2.71 × 10^–7^	8.82%	6
KEGG:05204	Chemical carcinogenesis - DNA adducts	KEGG	2.71 × 10^–7^	8.82%	6
KEGG:00980	Metabolism of xenobiotics by cytochrome P450	KEGG	2.79 × 10^–7^	8.22%	6
KEGG:04976	Bile secretion	KEGG	6.98 × 10^–7^	6.74%	6
KEGG:05207	Chemical carcinogenesis - receptor activation	KEGG	4.47 × 10^–6^	3.32%	7
KEGG:00830	Retinol metabolism	KEGG	4.89 × 10^–6^	7.35%	5
KEGG:00591	Linoleic acid metabolism	KEGG	5.38 × 10^–6^	13.79%	4
KEGG:00140	Steroid hormone biosynthesis	KEGG	0.00302	4.92%	3
KEGG:00590	Arachidonic acid metabolism	KEGG	0.00302	4.92%	3
KEGG:04933	AGE-RAGE signaling pathway in diabetic complications	KEGG	0.01062	3.00%	3

### Pharmaco-Immunomodulatory Agents Such as Tocilizumab, Ritonavir, and Glucocorticoids (Dexamethasone, Methylprednisolone, and Hydrocortisone), Affecting PXR Regulation, Involved in DPTS-Induced Liver Injury

**FIGURE 5:**
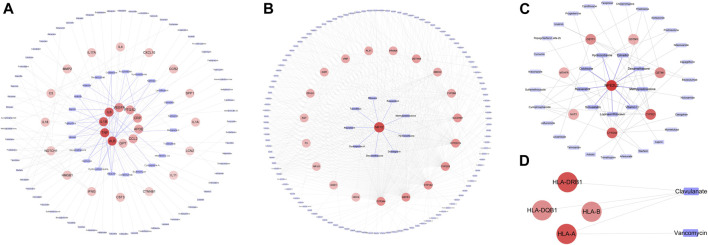
Association between Drugs in DPTS and DILI targets in MCODE sub-networks: *Cluster 1* **(A)** *, cluster 2* **(B)** *, cluster 3* **(C)** *, and cluster 4* **(D)**.

Pharmaco-immunomodulatory agents targeting inflammation in COVID-19 were the primarily involved drugs related to the inflammatory *cluster 1*. These drugs included the specific IL-6 antagonist of tocilizumab, non-specific agents (corticosteroids and interferons), macrolides, stains, and hydroxychloroquine/chloroquine. Additionally, immune-suppressive agents and some antibiotics also elicited anti-inflammatory properties (Figure 5A). Anti-inflammatory effects of these drugs might affect PXR expression and its regulated DM. These drugs were involved in DILI. Ritonavir and glucocorticoids (dexamethasone, methylprednisolone, and hydrocortisone) could activate PXR in *cluster 2* (Figure 5B). PXR regulated CYP3A4 and ABCB1 that metabolized and transported 41.2% (89/216) and 34.7% (75/216) of DPTS, respectively. Resveratrol interacted with PXR and inhibited CYP3A4 activity (Deng et al., 2014). The PK profiles of atazanavir, dolutegravir, tacrolimus, and cyclosporine A were found to be associated with PXR gene polymorphisms. Oleoylethanolamide and simvastatin could increase the expression of PPARA and its downstream CYP3A4 (de Keyser et al., 2013; Lama et al., 2020). In *cluster 3*, ritonavir and glucocorticoids (dexamethasone, methylprednisolone, and hydrocortisone) suppressed Nrf2 which regulated cellular antioxidant activity. Colchicine, vitamin D, simvastatin, estradiol, and resveratrol could activate Nrf2 and have antioxidant effects (Figure 5C). Additionally, treatment of clavulanate and vancomycin administered for secondary bacterial infection of COVID-19 were associated with HLA targets of *cluster 4* with a high risk of idiosyncratic DILI (Figure 5D).

Pharmaco-immunomodulatory agents such as tocilizumab and glucocorticoids (dexamethasone, methylprednisolone, and hydrocortisone) and ritonavir affect PXR regulation and cause DILI. Corticosteroids have a high likelihood score of DILI A with hepatic enlargement and steatosis or glycogenosis (nonalcoholic steatohepatitis). Log *p* values of corticosteroids ranged from 1.46 to 1.9, as listed in Table 4. Although lopinavir/ritonavir has a low likelihood score of DILI D/C, they have high lipophilicity (log *p* = 5.9/6, suggestive of extensive hepatic metabolism) and high daily doses (800/200 mg). These findings highlight their potential risk of DILI. Likewise, other anti-SARS-CoV-2-Virus drugs with log *p* ≥ 3 and a high daily dose ≥100 mg, e.g., hydroxychloroquine/chloroquine, may be also involved in DILI.

**TABLE 4:** Lipophilicity, route of administration, dose, and hepatotoxicity of drugs associated with inflammatory targets of *cluster 1*, PXR, PPARA, NRF2, and HLA targets.

DPTS		Lipophilicity/Log P	Route of Administration	Dose	Metabolic Organ	Likelihood Score of DILI	Patterns of DILI
Anti-SARS-CoV-2-virus	
Ribavirin		−1.8	PO	1.2–2.4 g q8h	Kidneys	E	Suspected
Nelfinavir		6	PO	—	Liver	D	H
Lopinavir		5.9	PO	400 mg/100 mg (Rito) q12 h, 14 days	Liver	D	H, C or M
Ritonavir (Rito)		6	PO	100 mg q12 h, 14 days	Liver	C	H, C; exacerbation of an underlying chronic hepatitis B or C
Atazanavir		5.6	PO	300/100 mg (Rito)	Liver	D	SAE, indirect HB, idiosyncratic; exacerbation of an underlying chronic hepatitis B or C
Chloroquine		4.6	PO	500 mg q12 h or qd, 5–10 days	Liver	D	SAE or jaundice
Hydroxychloroquine		3.6	PO	400 mg q12 h, 1 day, then 200 mg, q12 h, 4 days	Liver	C	SAE
Dolutegravir		2.2	PO	50 mg qd	Liver	D	SAE, H
Pharmaco- immunomodulatory							
IL-6 receptor antagonists	Tocilizumab	—	IV	400 mg or 8 mg/kg, 1–2 doses. Second dose 8–12 h after 1st dose	—	C	H
	Sarilumab	—	H	200 mg or 400 mg, 1 dose	—	E	SAE
Macrolides	Erythromycin	2.7	PO	4 g daily in divided doses	Liver	A	C, H (rare)
Interferon	Interferon alfa-2b	—	Nebulized	5 million units bid	—	A	SAE, C
Corticosteroids	Methylprednisolone	1.9	IV, PO	1 mg/kg qd (IV) 5 days, 40 mg qd 3 days, 10 mg qd 2d	Liver	A [HD]	H (methylp), hepatic enlargement and steatosis or glycogenosis; NASH; exacerbation of an underlying chronic hepatitis B or C
	Prednisolone	1.62	IV, PO	≥10 mg/day, 6 weeks			
	Prednisone	1.46	IV, PO	various doses and ≥0.5 mg/kg			
	Dexamethasone	1.9	IV, PO	20 mg qd (IV) 5 days, 10 mg qd 3days, 5 mg qd 2 days, 7d			
	Hydrocortisone	1.6	IV	50 mg or 100 mg q 6 h			
Colchicine		1	PO	0.5 mg bid 3 days, then 0.5 mg qd 27 days	Liver	E	Unlikely
Nafamostat		—	IV	10 mg qd or bid	Liver	—	—
Imatinib		3	PO	400 mg qd 14 days	Liver	B	SAE; exacerbation of an underlying chronic hepatitis B
Leflunomide		2.8	PO	LD: 50 mg q12 h 3times; 20 mg qd 10d	Liver	B	SAE; exacerbation of an underlying chronic hepatitis B
IL-1 receptor antagonists	Anakinra	1.1	IV, H	5 mg/kg q12 h; LD: 300 mg, 10 mg q6 h; 100 mg qd or q12 h (H)	Not available	C	H; exacerbation of an underlying chronic hepatitis B or C
	Canakinumab (IL-1beta)	—	SC	300 mg 1dose	—	E	suspected
JAK1/JAK2 inhibitor	Baricitinib	-0.5	PO	2 or 4 mg qd for 14 days	Liver	E	Unlikely
Anti-CD20	Rituximab	—	IV	375 mg/m^2^	—	A	SAE; exacerbation of an underlying chronic hepatitis B
Anti-VEGF	Bevacizumab	—	IV	500 mg	—	E	Unlikely
Anti-TNF	Infliximab	—	IV	50–200 mg	Reticuloendothelial system	A	H, C or M; exacerbation of an underlying chronic hepatitis B
C5 complement inhibitor	Eculizumab	—	IV	on trial and not determined	—	D	SAE
Immune-suppressive agents	Cyclosporine A	1.4	IV, PO	target at 83·2–374·4 nmol/L or up to 600–1050 nmol/L, 7-10 days or up to 21 days	Liver and intestinal tract	C	HB
	Tacrolimus	3.3	IV, PO	on trial and not determined	Liver	C	SAE
Stains	Simvastatin	4.68	PO	40 mg qd 14 days	intestinal wall, liver, and plasma	A	SAE
	Rosuvastatin	0.13	PO	40 mg qd 14 days	not extensively metabolized	B	SAE
Anti-platelet drugs
Aspirin		1.2	PO	100 mg qd at least 5 days	Liver	A [HD]	SAE; Reye syndrome
Warfarin		2.7	PO	5 mg qd, 2–4 days, followed by 2–10 mg qd	Liver	C	H, C or M
**Others**							
Celecoxib		3.53	PO	various doses	Liver	B	H, C or M; accompanied with hypersensitivity
Estradiol		4.01	PO	on trial and not determined	Liver and intestinal tract	—	—
Oleoylethanolamide		—		—	—	—	—
Resveratrol		—	PO	Various doses	Liver	E	unlikely
Vitamin D		7.5	multiple forms	Various doses	Liver and kidneys	E	Unlikely
Antibiotics against bacterial co-infections with SARS-CoV-2							
Community	Doxycycline	−0.7	PO	200 mg qd d1, 100 mg qd, d 2–5	Liver and gastrointestinal tract	B	H, C or M; accompanied with hypersensitivity
	Clavulanate (Amoxicillin)	−1.2	IV, PO	125 mg tid 5 days	Kidneys	A	C, H or M, Idiosyncratic
	Levofloxacin	−0.4	IV, PO	250–750 mg (PO) qd or 500 mg (IV) qd,7–14 days	Largely not metabolized	A	H, C and M; accompanied with hypersensitivity
Ward	Ampicillin (sulbactam)	−1	IM, IV	1.5–3 g q 6 h, 7–14 days	Not extensively metabolized	C	SAE; accompanied with SJS, TEN
	Meropenem	−2.4	IV	0.5–1 g q8h	Kidneys	D	SAE, C
	Vancomycin	−2.6	IV	0.5 g q6h or 1g q12 h	Kidneys	B	SAE accompanied with DRESS syndrome, SJS, TEN
	Cefotaxime	−1.4	IV	2g q6-8 h	Kidneys	—	—
	Daptomycin	−5.1	IV	4–6 mg/kg qd, 7–28 days	Not interact with CYP450s	C	SAE, H
	Tigecycline	1.1	IV	LD: 200 or 100 mg, 100 or 50 mg q12h, 5–14 days	Not extensively metabolized	E#	Suspected

**Abbreviations:** C, cholestatic; DILI, drug induced liver injury; DPTS, drugs of pharmacologic treatment against SARS-CoV-2; DRESS, drug reaction with eosinophilia and systemic symptoms; H, hepatocellular;; HD, high dose; IL, interleukin; IM, intramuscular; IV, intravenous; JAK, janus kinase; LD, loading dose; Log P, log value of octanol-water partition coefficient; M, mixed; NASH, nonalcoholic steatohepatitis; PO, oral; SAE, serum aminotransferase elevations; SARS-CoV-2, severe acute respiratory syndrome coronavirus 2; SC, subcutaneous; SJS, stevens johnson syndrome; TEN, toxic epidermal necrosis; VEGF, vascular endothelial growth factor; # in patients with acute porphyria and porphyria cutanea tarda.

## Discussion

In the present study, 1209 DPTSGs, including genes encoding PK and therapeutic targets of DPTS, were found to be associated with drug metabolism of CYP450s, nuclear receptor of PXR, and COVID-19 adverse outcome. Regulatory network of DPTS-induced liver injury contained inflammatory sub-network (*cluster 1*, MCODE score = 22.08), PXR-regulated drug metabolic sub-network (*cluster 2*, MCODE score = 8.421), NRF2-regulated oxidative sub-network (*cluster 3*), and HLA-related adaptive immune sub-network (*cluster 4*). In the primary DILI sub-network of *cluster 1,* IL6, IL1B, TNF, and CCL2 were among the top ten key targets and related to the COVID-19 adverse outcome pathwaya inflammation of CRS exacerbated DILI. Pharmaco-immunomodulatory agents, including tocilizumab that targets at IL-6 of CRS and glucocorticoids (dexamethasone, methylprednisolone, and hydrocortisone), and ritonavir affected PXR regulation. DDIs between these above drugs resulted in DPTS-induced liver injury. Moreover, ritonavir and glucocorticoids (dexamethasone, methylprednisolone, and hydrocortisone) suppressed antioxidant Nrf2, promoting DILI. Additionally, clavulanate and vancomycin, associated with HLA targets of *cluster 4*, had high risk of idiosyncratic DILI. Our results identified the key regulatory effects of PXR in DILI associated with CRS of COVID-19 and DDIs between some medications in the treatment of COVID-19 patients.

PXR, regarded as a master xenobiotic sensor and predominantly expressed in the liver, contributed to significant DDIs and DILI, leading to severe clinical ramifications (Sinz, 2013). In the absence of xenobiotics, above DM genes are expressed at the minimal basal levels. Activation of PXR by xenobiotics (drugs), induced the expression of a large number of genes in the hepatic DM system, including CYP3A4, CYP2E1, UGTs, ABCB1, leading to the detoxification of xenobiotics (Wang et al., 2014). Whereas, the augmented system is a double-edged sword for DILI in COVID-19 patients because it needs to convert parent drug into toxic reactive intermediates, e.g., ritonavir, to facilitate the detoxification of exogenous compounds. In addition, PXR played pleiotropic effects on DILI: this xenosensor could be inhibited by significant liver inflammatory response, attributing to CRS of COVID-19, via the crosstalk with NF-κB (Zordoky and El-Kadi, 2009; Kanan et al., 2021). The p65 subunit, increased by IL-6 and other released cytokines, interacted with the PXR partner RXRα to inhibit hepatic metabolism system (Moreau et al., 2008).

This PXR inhibition mediated inflammation-drug interactions via the decreased CYP450s activity such as CYP3A4 and increased substrate drugs to the supratherapeutic levels that might result in DILI. In the study of Lenoir, et al. (Lenoir et al., 2021), they observed that the metabolic ratios of CYP3A, CYP2C19, and CYP1A2 were significantly lower when the serum levels of CRP, IL-6, and TNF-α were significantly higher (*p values* < 0.045) in the patients suffering SARS-CoV-2 infection. CRP is an acute inflammatory protein synthesized by hepatocytes, and its production is mainly regulated by IL-6 (Del Giudice and Gangestad, 2018). Lopinavir trough concentration (C_trough_) was reached an extremely high level >20,000 ng/ml (six times the upper therapeutic limit), leading to the increased liver injury, in approximately 75% of the ICU patients receiving the halving dose (400/100 mg q24 h) with plasma CRP >200 mg/L (Lê et al., 2020). Consistent with these results, lopinavir C_trough_ levels (median of 13600 ng/ml with the standard 400/100 mg q12 h) were significantly correlated with CRP (Spearman correlation coefficient = 0.81) and approximately 2-fold higher in the hospitalized COVID-19 patients than in the HIV patients (7100 ng/ml) (Schoergenhofer et al., 2020). Additionally, the use of resveratrol which was an inhibitor of PXR could enhance the suppression of CYP3A4, CYP2C9, CYP2C19, and CYP1A2 enzymes (Hyrsova et al., 2019; Liu et al., 2021). In this state of impaired CYP450s, co-administration of remdesivir and simvastatin (both being the substrates of CYP3A4) should be avoided to prevent supra-therapeutic drug exposures and hepatotoxicity resulting from DDIs (Feingold, 2022). Furthermore, the patients’ susceptibility to DILI after supra-therapeutic drug exposures could be increased by liver inflammation due to CRS in COVID-19, attributing to ROS production via activation of NADPH oxidase (Maruf and O'Brien, 2014). The local liver inflammation might attribute to the proliferation or hyperplasia of Kupffer cells, which were the resident hepatic macrophages, as a consequence of systemic inflammation in COVID-19 patients (Nardo et al., 2021). In contrast, Marzolini, et al. (Marzolini et al., 2020) found that lopinavir C_trough_ was significantly lower when the IL-6 antagonist of tocilizumab was pre-administered (*p* < 0.001). These findings demonstrated that tocilizumab activated PXR with up-regulated CYP 3A4 expression by inhibiting IL-6 signaling.

However, PXR activation, induced by DDIs among pharmaco-immunomodulatory agents (anti-inflammation), ritonavir, and glucocorticoids (dexamethasone, methylprednisolone, and hydrocortisone), caused DILI. Increased CYP3A4 expression and abnormal lipid metabolism also contributed to DILI. PXR was activated as a result of the anti-inflammatory properties of pharmaco-immunomodulatory agents administrated to severe COVID-19 patients (the inflammatory phase) (El-Ghiaty et al., 2020). Tocilizumab increased the expression of PXR and decreased the IL-6 levels, affecting the PK of methotrexate which was the substrate of its downstream MRP2 (Zhou et al., 2021). Furthermore, PXR activation could be augmented by other co-administrated drugs including macrolides and hydroxychloroquine/chloroquine due to their inhibition of cytokines and chemokines (CCL2) production (Baralić et al., 2020; Rizk et al., 2020). PXR activation resulted in the accumulation of reactive metabolites and caused metabolic idiosyncratic DILI (Wang et al., 2014; Kobayashi et al., 2019; Wang et al., 2020). Increased PXR activation promoted the biotransformation of ritonavir into toxic M1 and M13 via CYP3A4 and resulted in oxidative stress and ER stress, ultimately leading to DILI. In a 52-year-old COVID-19 patient, administration of the two doses of 8 mg/kg tocilizumab (400 mg each) following a 15 days twice/day administration of lopinavir/ritonavir (400/100 mg) in combination with methylprednisolone, ceftriaxone, and azithromycin, resulted in acute liver injury (Muhović et al., 2020). In most reported cases, tocilizumab resulted in severe hepatic injury when it was used in combination with other potentially hepatotoxic drugs. PXR activation-dependent DDIs between tocilizumab and ritonavir supported our hypothesis that the hepatotoxic effect of tocilizumab was favored by accumulated use of lopinavir/ritonavir. Moreover, PXR activation of ritonavir increased fatty acid synthesis and induced mitochondrial dysfunction, highlighting the potential of DILI to cause hepatic steatosis. Besides anti-inflammatory effects, dexamethasone could directly activate PXR and induce hepatotoxicity (hepatomegaly) and lipid (triglycerides) accumulation. Dexamethasone-induced PXR activation increased fatty acids uptake and triglycerides accumulation, lipogenesis, decreased β-oxidation. It also promoted insulin resistance, and induced nonalcoholic steatohepatitis (NASH) (Cave et al., 2016). Methylprednisolone and hydrocortisone indirectly induced PXR gene expression via the glucocorticoid receptor pathway (Bhadhprasit et al., 2007). Oxidative stress and endoplasmic reticulum stress caused by ritonavir further promoted lipid accumulation resulting in NASH (Katsiki et al., 2016). Metabolic idiosyncratic DILI of ritonavir and hepatotoxicity (NASH) of glucocorticoids could be exacerbated by their interactions with pharmaco-immunomodulatory agents (DDIs) via PXR activation. In line with this, moderate microvesicular steatosis and mild lobular and portal activity were found in liver biopsy specimens of patients who died from COVID-19. Significant increase in the use of corticosteroids (24–120, *p* < 0.01) and immunosuppressants (3–89, *p* < 0.01; 91% tocilizumab) was observed in COVID-19 patients during their hospitalization (Cattaneo et al., 2020).

Furthermore, PXR regulation was accompanied by the suppression of Nrf2, an antioxidant response regulator, by glucocorticoids and lopinavir/ritonavir, aggravating DILI. Nrf2 regulates GSH production and the expression of GSTs and participates in ROS and xenobiotic (drugs) detoxification (Tonelli et al., 2018). Kratschmar et al. (Kratschmar et al., 2012) reported that glucocorticoids, acting through GR, suppressed Nrf2-dependent cellular antioxidant defenses in hepatic cells. Lopinavir/ritonavir inhibited Nrf2 expression and GSTs in mice livers (Hu et al., 2015). Colchicine, vitamin D, simvastatin, estradiol, and resveratrol could upregulate Nrf2 and decrease hepatic oxidative stress (Zhu et al., 2015; Abo El-Magd and Eraky, 2020; Awad et al., 2020; Liu et al., 2021). Clavulanate and vancomycin were associated with HLA-related sub-network, including HLA-B, HLA-DRB1, HLA-A, and HLA-DQB1. These HLA risk alleles were responsible for T-cell mediated-delayed hypersensitivity reactions, including immune idiosyncratic DILI (Redwood et al., 2018). Clavulanate was primarily associated with HLA-DRB1*15:01 and accounted for up to 17% of severe DILI cases (Andrade et al., 2006; Lucena et al., 2011). The risk of vancomycin-induced drug reaction with eosinophilia and systemic symptoms, accompanied by liver injury, was significantly high in patients with HLA-A*32:01 (Konvinse et al., 2019). Patients with COVID-19 receiving clavulanate or vancomycin in combination with lopinavir/ritonavir and glucocorticoids might need close monitoring or prior HLA testing to avoid the potential immune idiosyncratic DILI.

PXR-mediated ABCB1/P-gp and CYP2E1 were also involved in DPTS-induced liver injury. *In vitro* studies of Ambrus, et al., (Ambrus et al., 2021), highlighted the interactions of lopinavir/ritonavir, remdesivir, and chloroquine with ABCB1/P-gp, which were suggestive of the potential DDI. Because of the potential DDIs between remdesivir and P-gp inhibitors (chloroquine and amiodarone), a male patient with COVID-19 developed acute DILI (Leegwater et al., 2021). The reduced P-gp expression, mediated by PXR inhibition, in COVID-19 patients might increase the risk of DILI, associated with DDIs. Likewise, PXR inhibition could also induce cholestatic DILI, attributing to the decreased toxic bile acids metabolism (GSTs and UGTs) and elimination (ABCB1 and ABCC2) (Jonker et al., 2012; Cardoso et al., 2020). CYP2E1 genes might be activated by inflammatory cytokines of COVID-19 via NF-κB pathway, but the underlying ROS production would not be alleviated by the inhibited Nrf2 signaling. Dexamethasone inhibited the CYP2E1 expression. Acetaminophen-induced liver injury resulted from increased reactive metabolites and enhanced hepatic CYP2E1 activity (Ferron et al., 2020). Acetaminophen is commonly used to control fever and inflammation in COVID-19 patients. However, our results further demonstrated that there was not enough evidence to support this treatment.

This study has some limitations. Although bioinformatics analysis has been widely used in the advanced researches in biomedical sciences and drug design, there are the gaps between bioinformatics and system biology simulations and clinical practice. Firstly**,** drugs of pharmacological treatments in patients with concomitant primary diseases were not included. Of those patients, critically ill COVID-19 patients had a high risk of DILI, due to the primary hepatic dysfunction regularly and co-administration of multiple drugs, e.g., midazolam (sedative) and valproic acid (antiepileptic). In the complex pharmacotherapy, most of drugs were metabolized by CYP3A4 in the liver (Farrokh et al., 2018)**.** The reduced CYP3A4 activity increased the risk of severe adverse drug events and affected clinical outcomes. Whereas, oleoylethanolamide and simvastatin increased the CYP3A4 expression via PPARA (de Keyser et al., 2013; Lama et al., 2020). Secondly, because of DPTS metabolized by multiple DMEs, the complexity of DDIs enhanced the DILI risk. Lopinavir/ritonavir also induced the expressions of CYP1A2, CYP2C9, CYP2C19, and UGT1A1. Moreover, CYP1A2, CYP2C9, and CYP2C19 were the important isoenzymes involved in the metabolism of DPTS. Thirdly, some included drugs activating PXR would be without hepatotoxicity. Since PXR can be activated by a variety of structurally diverse drugs owing to its specific protein structure (large and flexible ligand-binding pocket) (Wang et al., 2020). Future studies are warranted to validate and explore the mediatory role of PXR in DILI associated with inflammation-drug and drug-drug interactions in COVID-19 patients who receive high-risk drugs.

## Conclusion

**FIGURE 6:**
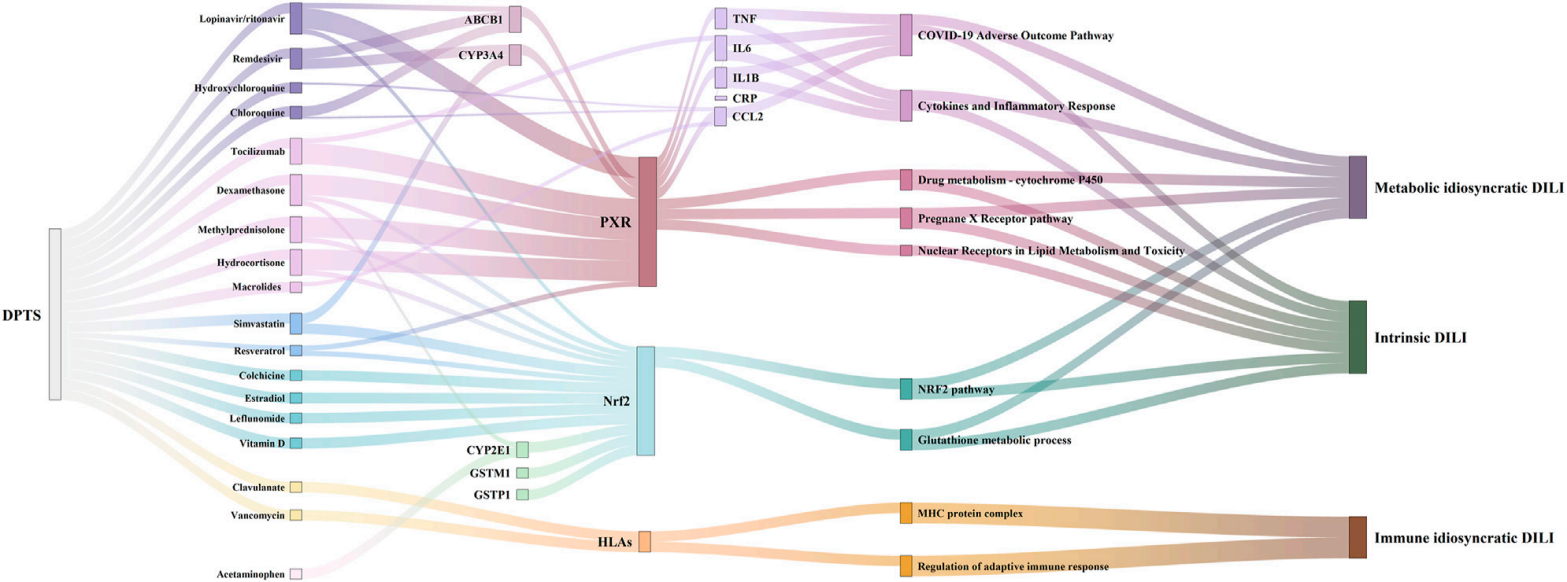
Potential mechanism underlying the DPTS-induced liver injury.

In conclusion, we provided in-depth insights to understand the mechanism underlying DPTS-induced liver injury (Figure 6): PXR regulation, associated with inflammation-drug and drug-drug interactions, mediated DILI in pharmacologic treatments for COVID-19. This study highlighted the cautious clinical decision-making for pharmacotherapy to avoid DILI, especially in critically ill COVID-19 patients.

## Data Availability

The original contributions presented in the study are included in the article/Supplementary Material, further inquiries can be directed to the corresponding authors.
